# High HTLV-I proviral load in patients with HAM/TSP and ATLL but not with other disorders

**DOI:** 10.1186/1742-4690-8-S1-A36

**Published:** 2011-06-06

**Authors:** Natalia Altamirano, Carlos Remondegui, Ariel Fridman, Mirta Remesar, María B Bouzas, Patricia Paradiso, Patricia Costantini, Ines Zapiola, Carlos Rocco, Paula Aulicino, Luisa Sen, Andrea Mangano

**Affiliations:** 1 Laboratorio de Biología Celular y Retrovirus, Hospital Garrahan, Ciudad Autónoma de Buenos Aires, Argentina; 2 Servicio de Infectología y Enfermedades Tropicales, Hospital San Roque, Jujuy, Argentina; 3 Laboratorio Central de Jujuy. Jujuy, Argentina; 4 Servicio de Hemoterapia, Hospital Garrahan, Ciudad Autónoma de Buenos Aires, Argentina; 5 Unidad de Virología, Hospital Muñiz, Ciudad Autónoma de Buenos Aires, Argentina; 6 Servicio de Hemoterapia, Hospital Durand, Ciudad Autónoma de Buenos Aires, Argentina; 7 Hospital Angel H. Roffo, Ciudad Autónoma de Buenos Aires, Argentina

## Background

HTLV-I proviral load (pVL) is proposed as a biomarker of disease progression. HTLV-I pVLs are higher in symptomatic compared to asymptomatic carriers. The aim of this study was to evaluate HTLV-I pVL in patients with HTLV-I associated diseases (HAM/TSP and ATLL) and other disorders (uveitis and pyoderma gangrenous–PG).

## Methodology

HTLV-I pVL was evaluated in 62 subjects; 52 asymptomatic and 10 symptomatic (5 HAM/TSP, 2 ATLL, 2 uveitis and 1 PG). HTLV-I pVL in PBMCs was estimated by a quantitative real-time PCR assay with SYBR Green, where HTLV-I pol gene is amplified in parallel with albumin gene as a normalizer. The limit of detection of the assay was of 400 copies of HTLV-I/10^6^ PBMCs (0.04%). Differences among groups where assessed by Mann-Whitney test.

## Results

Symptomatic subjects (median=5.02 log10 copies/10^6^ PBMCs, IQR=4.70-5.30) had significantly higher pVLs than asymptomatic carriers (median=4.11 log10 copies/10^6^ PBMCs, IQR=3.55-4.66, p=0.0015). HAM/TSP patients had pVLs between 5.00-5.73 log10 copies/10^6^ PBMCs (10-54%), ATLL patients had pVLs of 4.83 and 5.50 log10 copies/10^6^ PBMCs (7% and 33%, respectively); patients with uveitis had pVLs of ~4.00 log10 copies/10^6^ PBMCs (~1%) and PG patient had pVL of 4.56 log10 copies/10^6^ PBMCs (4%). Patients with HAM/TSP and ATLL had significantly higher pVL (median=5.25 log10 copies/10^6^ PBMCs) than those with other disorders (median=4.04 log10 copies/10^6^ PBMCs, p=0.017).

## Conclusion

HTLV-I pVL seems to be associated with pathogenic phenotype, being higher in subjects with HTLV-I associated diseases compared to those with other disorders.

